# Information about ADRs explored by pharmacovigilance approaches: a qualitative review of studies on antibiotics, SSRIs and NSAIDs

**DOI:** 10.1186/1472-6904-9-4

**Published:** 2009-03-03

**Authors:** Lise Aagaard, Ebba Holme Hansen

**Affiliations:** 1 Department of Pharmacology and Pharmacotherapy, Section for Social Pharmacy, Faculty of Pharmaceutical Sciences, University of Copenhagen, Denmark; 2 FKL-Research Centre for Quality in Medicine Use, Denmark; 3 The Danish Medicines Agency, Copenhagen, Denmark

## Abstract

**Background:**

Despite surveillance efforts, unexpected and serious adverse drug reactions (ADRs) repeatedly occur after marketing. The aim of this article is to analyse ADRs reported by available ADR signal detection approaches and to explore which information about new and unexpected ADRs these approaches have detected.

**Methods:**

We selected three therapeutic cases for the review: antibiotics for systemic use, non-steroidal anti-inflammatory medicines (NSAID) and selective serotonin re-uptake inhibitors (SSRI). These groups are widely used and represent different therapeutic classes of medicines. The ADR studies were identified through literature search in Medline and Embase. The search was conducted in July 2007. For each therapeutic case, we analysed the time of publication, the strengths of the evidence of safety in the different approaches, reported ADRs and whether the studies have produced new information about ADRs compared to the information available at the time of marketing.

**Results:**

79 studies were eligible for inclusion in the analysis: 23 antibiotics studies, 35 NSAID studies, 20 SSRI studies. Studies were mainly published from the end of the 1990s and onwards. Although the drugs were launched in different decades, both analytical and observational approaches to ADR studies were similar for all three therapeutic cases: antibiotics, NSAIDs and SSRIs. The studies primarily dealt with analyses of ADRs of the type A and B and to a lesser extent C and D, cf. Rawlins' classification system. The therapeutic cases provided similar results with regard to detecting information about new ADRs despite different time periods and organs attacked. Approaches ranging higher in the evidence hierarchy provided information about risks of already known or expected ADRs, while information about new and previously unknown ADRs was only detected by case reports, the lowest ranking approach in the evidence hierarchy.

**Conclusion:**

Although the medicines were launched in different decades, approaches to the ADR studies were similar for all three therapeutic cases: antibiotics, NSAIDs and SSRIs. Both descriptive and analytical designs were applied. Despite the fact that analytical studies rank higher in the evidence hierarchy, only the lower ranking descriptive case reports/spontaneous reports provided information about new and previously undetected ADRs. This review underscores the importance of systems for spontaneous reporting of ADRs. Therefore, spontaneous reporting should be encouraged further and the information in ADR databases should continuously be subjected to systematic analysis.

## Background

The thalidomide catastrophe around 1960 and additional experiences such as serious adverse drug reactions to high oestrogen oral contraceptives in the 1960s were probably the main reasons for the increasingly stringent requirements set to document development safety and the establishment of spontaneous reporting systems [1,2]. Over the years, the repeated occurrence of unexpected, serious adverse drug reactions (ADRs) has attracted wide professional and public attention, with the result that doubt has been cast on the effectiveness and quality of drug safety surveillance systems. The COX-2 scandal resulting in worldwide withdrawal of Vioxx^® ^(rofecoxib) from the market in 2004 is a recent example of an ADR case that emerged unexpectedly and took the world by surprise [3]. Several other ADR cases have been discovered after marketing; well known are fenfluramine and the risk of pulmonal hypertension, vigabatrine and visual field defects and tolcapone and the risk of liver toxicity [4-6]. The repeated occurrence of serious ADR cases after medicines have been released on the market questions the extent to which existing systems and methods for predicting ADRs are effective [7]. Information about the ADR profile of a new medicine appears from observations made during the clinical development process [8,9]. The gold standard for the design of these clinical trials is the randomised controlled clinical trial (RCCT) [8,9]. The RCCT was designed to measure efficacy rather than ADRs as outcome. The design of the RCCT as hypothesis testing in itself sets narrow limits for the detection of information about serious and unexpected ADRs due to the short treatment period, the relatively small number of carefully selected participants in the trial, fixed drug doses, and hospital settings that do not reflect the conditions under which the medicines are used after marketing [8,9]. Data on well-recognised, easily detectable ADRs may potentially be observed in RCCTs, but unknown, rare or long-term adverse effects are seldom detected in these trials due to the limitations of the RCCT. Detection of unknown or rare ADRs may include other pharmacovigilance designs, e.g. the spontaneous reporting systems, cohort or case-control studies [1,10-12]. This article aims to review ADRs reported by available ADR signal detection approaches and to explore which information about new and unexpected ADRs these approaches have detected.

## Methods

We selected three different therapeutic groups of medicines for review. The groups were characterised by different:

### a. Therapeutic groups

• Antibiotics for systemic use

• Non-steroidal anti-inflammatory drugs (NSAIDs)

• Selective serotonin re-uptake inhibitors (SSRIs)

### b. Market launch

Antibiotics were first marketed in the 1940s and NSAIDs in the 1960s, while SSRIs were not launched until the middle of the 1980s (internal documents, The Danish Medicines Agency).

### c. ADR profiles

The therapeutic categories present different ADR profiles due to their specific pharmacological characteristics and functions.

### Literature search

**Figure 1:**
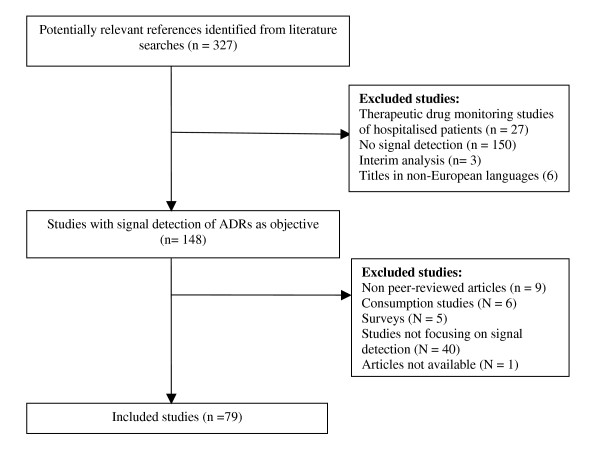
**Flow chart of the study selection process for the cases**.

Studies were identified through Medline (from 1966) and Embase (from 1989) using the following MESH terms: serotonin re-uptake inhibitors, anti-inflammatory agents, non-steroidal, anti-bacterial agents, adverse drug reaction reporting systems, pharmacoepidemiology and the key words: adverse drug reactions and information in combination. The literature search was conducted in July 2007 without language restriction. Studies written in non-European languages were later excluded. To be considered relevant for this review, articles had to be empirical in origin and focus on signal detection. Titles and abstracts of the search results were screened and relevant articles identified. The reference lists of included publications were hand-searched for possible additional relevant studies. Non peer-reviewed articles or unpublished observations were not considered. A flow chart of the study selection process for the therapeutic cases is illustrated in figure 1.

### Characteristics of the included studies

We developed a taxonomy inspired by general guidelines for pharmacoepidemiological research to analyse the studies systematically [13]. The taxonomy covers the following characteristics: publication year, design, method, explored medicine and adverse drug reactions, geographic setting, sampling period, sample size, outcome measures and results. We extracted and compared the results of published empirical studies in which various signal detection methods were used. Extracted information was entered into data sheets, one for each article. Data were extracted and handled by the first author and checked by the second author.

### Analyses

For each of the three selected therapeutic groups, we analysed the time of publication, the strengths of the evidence in the different approaches, reported ADRs and whether the studies had produced new information about ADRs compared to the information available at the time of marketing.

#### Classification of the tested/detected ADRs

For each included literature reference, the ADRs tested or detected via the various signal detection approaches were classified according to Rawlins' classification system [14]. An overview of the classification system is shown in table 1. The reported/detected ADRs were also classified according to System Organ Classes in keeping with MedDRA terminology [13].

**Table 1:** Rawlins' classification system of ADRs

Type	Definition
A	Dose-dependent ADRs related to the pharmacological effect of the drug:
	• Increased pharmacological effect
	• ADRs that occur secondarily to the desired pharmacological effect
	• ADRs due to other well known pharmacological effects

B	Sensitivity reactions – not dose-dependent
	• Allergic reactions
	• Idiosyncratic reaction

C	Long-term ADRs
	• Carcinogines
	• Teratogenes
	• Chronic organ damage

D	Drug-drug interactions
	• Pharmacodynamic
	• Pharmacokinetic
	• Non-classifiable

#### Classification of applied approaches

The explored approaches were classified into analytical or observational approaches according to Strom's definitions [13]. Case-control and cohort studies are classified as analytical methods, while spontaneous reporting, case series/case reports and PEM studies are observational [13].

#### Time of publication

For each therapeutic group, we analysed whether there was a connection between time of publication and the applied study design.

#### Strength of evidence

Evidence-based medicine operates with an evidence hierarchy for evaluating the quality of the various study designs used for therapeutic studies [13]. At the top of this hierarchy are the meta-analyses (level 1), followed by RCCTs at the second level and other controlled trials at the third level. Cohort studies are placed at the fourth level, followed by case-control studies at the fifth level. At the bottom of the evidence hierarchy are cross-sectional surveys (level 6) and anecdotal case reports (level 7) [13].

## Results

The literature search identified 327 potentially relevant references for all three therapeutic groups, 149 of which were selected from the titles and abstracts and further screened for relevance. Eventually 79 references were included in this analysis. A flow chart of the selection and exclusion process is illustrated in figure 1. The included studies were distributed on the three therapeutic cases as follows: antibiotics: 23 studies; NSAID: 35 studies; SSRI: 20 studies. One reference was not accessible.

### ADR detection approaches applied

Table 2 provides an overview of the categorisation of the designs used in the included studies and their rank in the evidence hierarchy [13]. As the table indicates, the majority of the included studies dealt with analyses of data reported in Prescription Event Monitoring (PEM) programs and ADRs reported to national ADR databases, approaches ranking at levels six and seven in the evidence hierarchy.

**Table 2:** The analysed studies categorised by study design

**Study design**	**Rank in evidence hierarchy**		**Therapeutic** **cases**		
		**Antibiotics**	**NSAIDs**	**SSRIs**	**Total**

Cohort	4	5	4	1	10

Case control	5	2	3	2	8

PEM*	6	2	9	4	15

National ADR databases	7	7	14	11	32

Case series	7	2	3	1	6

Case reports	7	5	2	1	8

**Total number of studies**		**23**	**36**	**20**	**79**

*Prescription Event Monitoring Studies

### Study characteristics

Tables 3, 4 and 5 display the characteristics and descriptions of the analysed studies for each therapeutic case [15-92]. The tables show that the studies primarily dealt with analyses of ADRs of the type A and B, and to a lesser extent C and D. The evidence level of ADRs varied widely; some of the ADRs were documented in both the analytical and observational studies, others in only one of the designs.

**Table 3:** Characteristics of studies of the occurrence of ADRs related to antibiotics use

**Reference**	**Setting**	**Medicines**	**ADRs**	**Sampling period**	**Sample size**	**Outcome** **measures**	**Results** **(95% CI)**	**Type of ADRs**
**Case control studies**								

Czeizel 1999 [15]	HU	Erythromycin	Teratology	1980–1996	113 cases/38,151 controls	OR	1.1; 0.5–2.3	C

Seeger 2006 [16]		Fluoroquinolones	Achilles tendon rupture	1997–2001	947 cases/ 18,940 controls	OR	1.2; 0.9–1.7	B

**Cohort studies**								

Chysky 1991[17]	DE	Ciprofloxacin	Not specified	44 days	634 patients	% ADRs	Different categories reported	A/B

Derby 1993 [18]	AU	Flucloxacillin	Cholestatic hepatitis	45 days	132,087 patients	PRR/100,000 users	7.6; 3.5–13.9	B

Jick 1994 [19]	AU	Flucloxacillin	Cholestatic hepatitis	1991–1992	77,552 patients	PRR/ 100,000 users	6.5; 2.7–15.1	B

Derby 1993b [20]	AU	Erythromycin	Cholestatic hepatitis	-	366,064 patients	PRR/100,000 users	3,6; 1.9–6.1	B

Heymann 2007 [21]	Israel	Penicillins	Pemphigus	1997–2001	150,000 patients	OR	2.03; 1.56–2.64	B

**PEM**								

Clark 2001 [22]	UK	Fluoroquinolones	Cardiovascular events	1988–1991	36,410 patients	CRR (crude relative risk)	Atrial fibrillation: 1.0; 0.02 – 8.92	B

Inman1994 [23]	UK	Fluconazole	All	1988–1989	15,015 patients	Frequencies	Different categories reported	A

**National ADR databases**								

Polimeni 2006 [49]	Sicilian	Antibacterials	All	1998–2002	1585 cases	ADRs	Different categories reported	A

Sachs 2006 [24]	DE	Fluoroquinolones	Anaphylaxis	1993–2004	204 cases	PRR > 2	Moxifloxacin: 2.1; Ofloxacin: 2.3 Ciprofloxacin: 2.3 Levofloxacin: 2.0	B

Fleisch 2000 [25]	CH	Levofloxacin	Tendinopathy	1986–1999	19 cases/460 non-cases	Reporting rate	Different categories reported	B

Leone 2003 [26]	IT	Fluroquinolones	Not specified	1999–2001	432 cases/ 10,011 non cases	Reporting rate	Different categories reported	A

Pierfitte 2000 [27]	FR	Sparfloxacin	Phototoxicity	1994–1996	371 cases	RtR/1000 patients	0.4	B

Frothingham 2005 [28]	US	Gatifloxacin	Glucose homeostatis abnormalities	1997–2003	453 cases/ 1427 non cases	Reporting rate/10^7 ^prescriptions	477	A

Hedenmalm 1996 [29]	SE	Fluorquinolones	Sensory disturbances	1965–1993	37 cases	ADRs	Different categories reported	A

**Case series**								

Abouesh 2002 [30]	-	Fluorquinolones Macrolides	Mania	-	102 cases	Case review	Case review	B

Smith 2005 [31]	-	Doxycycline Minocycline	ADRs	1966–2003	130 cases	Incidences	Doxycycline: 0–61% Minocycline: 11.7 – 83.3%	A

**Case reports**								

Hällgren 2003 [32]	-	Ciprofloxacin	Steven-Johnson syndrome	1988–2000	8 cases	IC pr. 100,000 patients	0.045	B

Warner 2000 [33]	-	Clarithromycin	Acute Psychotic Stress	-	1 case	Causality assessment	Possible	A

ADRAC 1992 [34]	-	Flucloxacillin	Cholestatic hepatitis	-	1 case	Case review	Case review	B

Greco 1997 [35]	-	Clarithromycin	Glossitis, stomatitis, black tongue	-	1 case	Case review	Case review	B

Björnsson 1996 [36]	-	Doxycycline	Liver reactions	1966–1995	23 cases	Causality assessment	Likely (n = 3) Possible (n = 8)	B

**Table 4:** Characteristics of studies of the occurrence of ADRs related to NSAID use

**Reference**	**Setting**	**Medicines**	**ADRs**	**Sampling period**	**Sample size**	**Outcome measures**	**Results** **(95%CI)**	**Type of ADRs**
**Case control** **studies**								

Hernandez-Diaz 2001[37]	UK	NSAIDs	Gastrointestinal events	1993–1998	2,105 cases/ 11,500 controls	OR	1.8; 1.3 – 2.4.	A

Mockenhaupt 2003 [38]	DE/US	NSAIDs	Steven-Johnson syndrome	1989–1995	245 cases/ 1147 controls	PRR	34, 95; 11–105	B

Lacroix 2004 [39]	FR	NSAIDs	Liver injury	1998–2000	88 cases/ 178 controls	OR	Women: 6.49; 1.67–25.16 Men: 1.06; 0.36–3.12	B

**Cohort studies**								

Lipworth 2004 [40]	DK	Ibuprofen	Mortality	1989–1995	113,538 patients	SMR (standard mortality rate)	1.21; 1.19–1.24	A/B

Ashworth 2004 [41]	CA	Diclofenac Naproxen Arthrotec	Mortality	1991–1994	18,424 patients	OR	Arthrotec: 1.4; 0.9–2.1. Diclofenac: 2.0; 1.3–3.1. Naproxen: 3.0; 1.9–4.6	A/B

Morant 2004 [42]	UK	NSAIDs	Gastrointestinal haemorrhage	1987–2001	628000 patient year	PRR	0.84; 0.60 – 1.17	A

Martin 2000 [43]	UK	Meloxicam	Gastrointestinal events	1996–1997	19,087 patients	Events/ 1000 patient-months of exposure	Dyspepsia: 28.3 Gastrointestinal haemorrhage: 0.4	A + B

**National ADR databases**								

Lugardon 2004 [44]	FR	COX-2 inhibitors	Oeso-gastro-duodenal events:	2000–2002	505 cases/ 2,525 non-cases	OR	14.9; 9.3–23.7	A

Durrieu 2005 [45]	FR	COX-2 inhibitors	Arterial hypertension	2000–2003	34 cases	OR	3.3; 1.6–6.9.	A

Clinard 2004 [46]	FR	NSAIDs	Excess risk of adverse drug reactions	1995–1999	3983 cases/ 54,583 non- cases	OR	Different categories reported	B

Brinker 2004 [47]	US	COX-2 inhibitors	Hypertension	< 2002	34 cases	Reporting rate/10^6^person years	Rofecoxib: 5.0 Celecoxib: 1.3	A

La Grenade 2005 [48]	US	COX-2 inhibitors Meloxicam	Steven-Johnson syndrome Toxic Epidermal Necrolysis	< 2004	123 cases	Reporting rate/10^6^person years	Valdecoxib: 49 Celecoxib: 6 Rofecoxib: 3	B

Polimeni 2006 [49]	Sicilian	NSAIDs	All	1998–2002	1585 cases	PRR	Hepatitis: 14.20 Vasculitis: 7.72 Hypertension: 15.40	B

Conforti 2001 [50]	IT	NSAIDs	Gastrointestinal events	1996–1999	705 cases/ 10,608 non cases	% ADRs	Nimesulid: 10.4 Diclofenac: 21.2 Ketoprofen: 1.7 Piroxicam: 18.6	A

Ahmad 2002 [51]	US	COX-2 inhibitors	Renal failure	1969–2000	Celecoxib: 122 cases Rofecoxib: 142 cases	Case review	Case review	A

Puijenbroek 2000 [52]	NL	NSAIDs Diuretics	Drug interactions	1990–1999	305 cases/ 9517 non cases	OR	OR: 2.0, 1.1–3.7	D

Lapeyre-Mestre 2004 [53]	FR/ES	NSAIDs	Hepatic events	1982–2001	29,486 cases	OR	Different OR calculated for NSAIDs.	B

Leone 1999 [54]	IT	Nimesulide	Renal impairment	1988–1997	11cases/ 7438 non cases	Causality assessment	Possible (n = 6) Probable (n = 4) Certain (n = 1)	A

Brown 1998 [55]	UK	Tiaprofenic acid	Cystitis	1981–1996	221 cases/ 1327 non cases	ADRs/10^5 ^prescriptions	1991: 4.2 1992: 5.9 1993: 4.2 1994: 34.4 1995: 18.5 1996: 6.5	B

Verrico 2003 [56]	US	COX2-inhibitors	Not specified	1999–2002	24 cases	Causality assessment	Possible (n = 29) Probable (n = 16)	A

Kahn 1997 [57]	US	NSAIDs	Necrotizing soft tissue infections	1969–1995	33 cases	Case review	N = 26	C

**PEM**								

Layton 2004a [58]	UK	Celecoxib	Not specified	2000	17,458 patients	IDs (event incidence densitites)	Dyspepsia = 25.4 Abdominal pain = 10.6	A + B

Layton 2003b [59]	UK	Celecoxib Meloxicam	Not specified	1996–1997	34,355 patients	PRR	Different categories reported	A

Layton 2003c [60]	UK	Rofecoxib	Not specified	2000	15,268 patients	Event rate pr. 1000 patient months exposure	76 upper GI bleedings and 101 thromboembolic events	A + B

Layton 2004d [61]	UK	Rofecoxib	Exacerbation of colitis	1999	15,268 patients	IRR	5.8; 2.7–11.3	A

Kasliwal 2005 [62]	UK	COX-2 inhibitors	Gastrointestinal + thromboembolic events	1999–2000	32,726 patients	PRR	GI: 1.21; 1.09 – 1.36. Thromboembolic: 1.04; 0.50 – 2.17.	A + B

Layton 2003e [63]	UK	Rofecoxib Meloxicam	Thromboembolic events	1996–1997	34,355 patients	PRR	1.68; 1.15 – 2.46.	A

Layton 2003f [64]	UK	Rofecoxib Meloxicam	Upper GI events	1996–1997	34,355 patients	IR	0.71; 0.65 – 0.79.	A

Layton 2006g [65]	UK	COX-2 inhibitors	Serious skin reactions	1999–2000/	52,644 patients	IR/1000 patient-months	IR: 0.019	B

Layton 2003h [66]	UK	Celecoxib Meloxicam	Gastrointestinal events	1996–1997	36,545 patients	PRR	0.77; 0.69 – 0.85.	A

**Case series**								

Onder 2004 [67]	-	NSAIDs	Psychiatric ADRs	1965–2003	27 reports with data on 453 cases	Risk factors	Age, psychiatric disorders, parturients	B

Fraunfelder 2006 [68]	-	NSAIDs	Ocular ADRs	-	569 cases	Reported ADRs	Blurred vision, conjunctivitis, visual hallucinations	B

Zimer 2007 [69]	DE	Valdecoxib	Cutaneous adverse reactions	2002–2005	5 cases	Case review	Erythematous, facial edema, dyspnea	B

**Case reports**								

Hunter 1999 [70]	-	Bromfenac	Hepatic Failure	-	1 case	Causality assessment	Related	B

ADRAC 1998 [71]	-	Diclofenac Indomethacin Mefenamic acid	Closure of fetal ductus arterious	-	3 cases	Case review	Case review	C

**Table 5:** Studies of the occurrence of ADRs related to SSRI use

**Reference**	**Setting**	**Medicines**	**ADRs**	**Sampling period**	**Sample size**	**Outcome measures**	**Results** **(95% CI)**	**Type of ADRs**
**Case-control studies**								

Schillevoort 2002 [72]	NL	SSRIs	Extrapyramidal Syndromes (EPS)	1985–1999	41cases/1,264 controls	OR	2.2; 1.2–3.9	A

Movig 2002 [73]	NL	SSRIs	Hyponatraemia	1990–1998	203 cases/608 controls	OR	3.96; 1.33 – 11.83	A

**Cohort study**								

Bell 2006 [74]	US	Fluoxetine	Testosterone levels	-	14 patients	Testosterone level	No changes	B

**National ADR databases**								

Trenque 2002 [75]	FR	SSRIs	Withdrawal syndrome	< 2000	60 cases/166,327 non cases	OR	5.05, 3.81–6.68.	A

Gony 2003 76]	FR	SSRIs	Extrapyramidal Symptoms	1995–2000	9 cases	OR	2.18; 0.47–11.35	A

Hedenmalm 2006 [77]	SE	SSRIs	Alopecia	< 2004	27 cases	IC	Sertraline = 1.63, 0.85–2.41 Citalopram = 1.22, 0.97–1.47	B

Goldstein 1997 [78]	-	Fluoxetine	First-trimester exposure on newborns	< 1996	796 cases	Rate %	5.0	C

Spigset 2003 [79]	SE	Nefazodone	Hepatic injury	< 2002	27,542 cases/ 2830764 non cases	IC	0.42, 0.12–0.72	B

Khan 2003 [80]	US	SSRIs	Suicide	1985–2000	77 cases/48,277 non cases	Suicide rate	0.59, 0.31 – 0.87	A

Egberts 1997 [81]	NL	SSRIs	Non-puerperal lactation	1986–1996	38cases/14,439 non cases	OR	2.7; 6.4–25.4	A

Kvande 2001 [82]	NO	SSRIs	Pancreatitis	< 2000	160 cases	No. of cases	160 cases	B

Stahl 1997 [83]	SE	SSRIs	Withdrawal reactions	< 1995	49, 393 cases	Number of reports/10^6^/ DDD	Paroxetine = 1.9 Sertraline = 2.1 Fluoxetine = 0.48	A

Spigset 1999 [84]	SE	SSRIs	Not specified	1965–1997	1202 cases	ADRs	Different categories reported	A + B

Sanz 2005 [85]	SE	SSRIs	Neonatal withdrawal syndrome	1968–2002	102 cases	IC	Paroxetine = 4.07 Sertraline = 1.20 Citalopram = 1.92 Fluoxetin = 1.07	C

**PEM**								

Price 1996 [86]	UK	SSRIs	Withdrawal reactions	1987–1992	50,150 patients	Reports/ 1000 prescriptions	Paroxetine = 0.3 Sertraline = 0.03 Fluvoxamine = 0.03 Fluoxetine = 0.002	A

Layton 2001 [87]	UK	SSRIs	Abnormal bleeding	1986–1998	135,754 patients	PRR	Day 1–30 = 1.38 Month 2–6 = 1.17	A

Edwards 1994 [88]	UK	Fluvoxamine	All	1987–1988	10,401 patients	Incidences		A

MacKay 1997 [89]	UK	SSRIs	All	1988–1991	56,145 patients		Nausea, vomiting, withdrawal symptoms	

**Case series**								

de Abajo 2006 [90]	-	SSRIs Venlafaxine	Bleeding Disorders	1988–2003	1,651 cases/ 10,000 controls	PRR	3.0, 2.1–4.4	A

Gram 1999 [91]	DK	SSRIs	Bleeding Thrombocytopenia	-	8 cases	-	Case review	A + B

**Case report**								

Demers 2001 [92]	-	Fluvoxamine	Serotonin syndrome	-	1 case	-	Case review	A

### Data sources

Case-control studies were carried out on data from various national registers and/or data from spontaneous ADR databases, physicians' databases such as the General Practitioners' database in the UK and Health Insurance Databases [15,16,37,38,72,73]. The studies were reported in the literature from the mid-1980s to the end of the 1990s. Cohort studies analysed ADR data collected from the mid-1980s to the end of the 1990s. The cohort studies varied in size from less than 20,000 patients to between 20,000–50,000 and more than 100,000 patients [17,19,21,40,41]. The PEM studies were conducted in the UK at the Drug Safety Unit in Southampton, and were based on data collected from the mid-1980s to the end of the 1990s [22,23,58-66,86-89]. Studies analysing spontaneously reported ADRs were conducted on large spontaneous reporting databases such as the French, American, British and the Uppsala Monitoring Centre WHO database [44-48,51,55-57,62,74-76,79,82,83,85].

### Design and historical perspective

The antibiotic studies were published from 1990 and onwards, most of them from 1995. Cohort studies were published during 1990–1994, while the PEM studies, spontaneous reporting, case reports/case series primarily were published after 1995. The majority of the NSAID studies were published after year 2000. The SSRI studies were published from 1990 to present, most of them from 1995 to 2005. Table 6 shows the distribution of the analysed studies by type of approach, therapeutic case, and time of publication. For all therapeutic cases, data were collected and the studies published a long time after the drugs were first marketed. Despite the decades of difference in market launches for the therapeutic cases, the studies are mainly published from the end of the 1990s and on. Data were collected earlier.

**Table 6:** Number of studies categorised by number, design and time of publication

**Year of publication**	**< 1990**	**1991**	**1992**	**1993**	**1994**	**1995**	**1996**	**1997**	**1998**	**1999**	**2000**	**2001**	**2002**	**2003**	**2004**	**2005-**
**Study design**																

**Antibiotics**																

Case control studies										1						1
Cohort studies		1		3												1
National ADR databases							1				2			1		2
PEM*					1							1				
Case series													1			1
Case reports			1				1	1			1			1		

**NSAIDs**																

Case control studies											1	1		1	1	
Cohort studies											1				3	
National ADR databases								1	1	1	1	1	1	1	4	3
PEM*														5	2	2
Case series															1	2
Case reports									1	1						

**SSRIs**																

Case control studies													2			
Cohort studies																1
National ADR databases								3		1		1	1	3		2
PEM*					1		1	1				1				
Case series										1						1
Case reports												1				

*Prescription Event Monitoring Studies

### Explored and detected ADRs

#### Antibiotics

ADRs from newer types of antibiotics, such as fluoroquinolones, have been reported much more frequently in the literature than ADRs from the older antibiotics, such as penicillins and macrolides [15-17,19,20,22-24,26,29,33,34,36]. The studies explore a possible risk between the use of antibiotics and the risk of liver, cardiovascular, CNS and dermatological ADRs [18-20,22,24,27,29,30,32,33,36]. Three cohort studies documented a correlation between cholestatic hepatitis and the use of flucloxacillin [18-20]. Increased risk of palpitation from the use of norfloxacin compared to ciprofloxacin/ofloxacin was demonstrated [22]. Cohort studies further demonstrated a risk of pemphigus related to penicillins, liver injury related to flucloxacillin and erythromycin [18-21]. CNS and dermatological ADRs from treatment with antibiotics have been reported rarely and on the case report level [30,32,33]. New information about ADRs was only produced by case reports: acute psychotic stress and glossitis/black tongue [34,35].

#### NSAIDs

Studies explored the risk of gastrointestinal [37-44,50] and dermatological ADRs as well as the development of liver and kidney toxicity which are well known ADRs associated with NSAIDs and their pharmacological characteristics[38,39,48,51,53,54,57,65,69,70]. The studies were generated after the launch of COX-2 inhibitors in the mid-1990s. A case-control study documented increased risk of developing dermatological ADRs of the type Steven-Johnson Syndrome and toxic epidermal necrolysis as did spontaneously reported ADRs [38,48]. A case-control study documented hepatic injury related to the use of NSAIDs, as did spontaneously reported ADRs, while renal injury and hypertension was documented in spontaneous reports and thromboembolic events in a PEM study [39,45,47,51,53,54,63,70]. With the exception of case reports, the approaches used did not produce information about ADRs that had not been reported previously.

#### SSRIs

Studies explored the risk of extrapyramidal symptoms, withdrawal syndromes and serotonin syndrome with the use of SSRIs, other ADRs investigated were: changes in testosterone and natrium level, alopecia, liver injury and bleeding. ADRs reported only via spontaneous reports are first-trimester exposure on newborns and neonatal withdrawal syndrome, hepatic injury and pancreatitis, suicide, non-puerperal lactation and serotonin syndrome [72-81,79,83,85,86,90-92]. With the exception of case reports, the approaches used did not detect new ADR signals that had not been reported previously [90-92].

### Information about ADRs reported across approaches

#### Analytical

The approaches produced information about ADR risks compared to placebo or similar drugs as either odds ratios (OR), proportional reporting ratio (PRR) estimates, incidences (IC) and frequencies of ADRs. These parameters are built into the design and based on previous information or hypothesis. The studies were conducted on various patient populations, various medicines within the individual sub-groups, and different types of ADRs, different outcome measures, data sources and time periods. The purpose of the approaches made it possible to adjust the ADR estimate for known confounders and risk factors.

#### Observational

The approaches produced information about ADRs as estimates (OR, PRR, IC) or as single observations compared to placebo/similar medicines. Case reporting was the only approach that contributed new information about new ADRs in all three therapeutic cases.

## Discussion

This review has several main findings:

First, analytical approaches ranging higher in the evidence hierarchy provided information about risks of already known or expected ADRs, while information about new and unknown ADRs was detected by case reports only, which range at the lowest level in the evidence hierarchy. Second, the studies primarily dealt with analyses of ADRs of type A and B, and only a few studies analysed type C and D. Third, similar approaches, both analytical and observational, were applied to all therapeutic cases. Fourth, the ADR cases provided similar results with regard to detecting new ADRs despite their connection to different time periods and organs attacked.

### Methodological quality and capability of approaches

There is a general lack of standards in the field of ADRs, particularly because many ADRs are not detected until after marketing and the studies are based on selected patient groups, which makes it difficult to generalise the results to other patient groups. As previously argued in the literature, testing specific hypotheses in the analytical approaches makes it difficult to capture information about new and unknown ADRs [13]. Despite the fact that these types of studies rank high in the evidence hierarchy, the weaker design of the observational studies makes them more suitable for discovering previously undetected ADRs. Healthcare professionals have conventionally considered cohort and case-control studies to be well suited for post-marketing surveillance of ADRs, despite their lack of randomisation and lower position in the evidence hierarchy, level 4 and 5 respectively [14]. These studies primarily detected/analysed ADRs of type A and B and less frequently type C and D [14]. Thus, the approaches are not designed and therefore are not suitable for predicting new information about other ADRs that have not previously been detected or ADRs of the type C or D [14]. Case reports have provided data about patients, suspected ADRs, medicines involved and so on, but this information is often anecdotal in nature and collected retrospectively. However, it is interesting that despite their low rank in the evidence hierarchy, these reports provide new information about rare and previous undetected ADRs. Case reports may serve as whistleblowers, thereby initiating larger systematic analyses of patient populations or registering data to quantify the risk. A large majority of spontaneously reported ADRs are stored in databases hosted by regulatory agencies. Information about these observations is typically only released to the public in the form of press releases, insertions in product information or messages in national bulletins. If all these signals were published in the scientific literature or made public on the web pages of regulatory agencies, the number of spontaneous reports/case series would probably have been larger and added to the relative dominance of this design [93,94]. The results confirm that spontaneous post-marketing reporting of ADRs is of great importance and that regulatory agencies must continue to encourage spontaneous reporting of ADRs [93,94].

### Alternative signal detection approaches

New ADR signals are often documented by only a small number of case reports, and systematic inclusion of data mining procedures in assessment of new ADR signals would probably contribute to earlier detection and quantification of serious ADR signals [95,96]. However, data mining was not applied in the three therapeutic cases studied here. Examples of data mining are cumulative techniques, time scans and Poisson methods, proportional reporting ratios (PRRs) and Bayesian data mining [97]. These methods assess how much the observed reporting frequency of a given drug-event combination deviates from that expected, given statistical independence between drug and event. Methodological and practical experiences with data mining in signal detection are limited [97,98].

### Strengths and limitations of the study

The objective of this review was to analyse which information signal detection approaches have produced about new ADRs in selected and published therapeutic cases, rather than to perform a systematic review of the entire body of ADR literature covering all therapeutic groups. The choice of widely different therapeutic cases and the similar results obtained across therapeutic cases make us believe that the results qualitatively reflect the general, published experience on ADRs based on signal detection approaches. Findings across therapeutic cases were similar with respect to methodological approaches and time of publication, despite the fact that ADRs differed in nature and affected different organs. Although antibiotics have been marketed since the 1940s, it was not possible to search for literature before the mid-1960s due to the limitations of current databases. Lack of consistency in reporting ADRs, different methodologies used in the studies and their impact on the results are difficult to evaluate in this review.

## Conclusion

Although the medicines were launched in different decades, approaches to the ADR studies were similar for all three therapeutic cases: antibiotics, NSAIDs and SSRIs. Descriptive as well as analytical designs were applied. Despite the fact that the analytical studies rank higher in the evidence hierarchy, only the descriptive case reports/spontaneous reports provided information about new and previously undetected ADRs. This review underscores the importance of systems for spontaneous reporting of ADRs. Therefore, spontaneous reporting should be encouraged further and the information in ADR databases should continuously be subjected to systematic analysis.

## Competing interests

The authors declare that they have no competing interests.

## Authors' contributions

LA and EHH designed the study, analysed the data and wrote the various drafts of the manuscript. LA did the sampling and literature search. Both authors read and approved the final version of the manuscript.

## Acknowledgements

We thank The Danish Medicines Agency and the Hørslev Foundation for their financial support of the study.

## Pre-publication history

The pre-publication history for this paper can be accessed here:


